# Soy Improves Cardiometabolic Health and Cecal Microbiota in Female Low-Fit Rats

**DOI:** 10.1038/s41598-017-08965-0

**Published:** 2017-08-23

**Authors:** Tzu-Wen L. Cross, Terese M. Zidon, Rebecca J. Welly, Young-Min Park, Steven L. Britton, Lauren G. Koch, George E. Rottinghaus, Maria R. Cattai de Godoy, Jaume Padilla, Kelly S. Swanson, Victoria J. Vieira-Potter

**Affiliations:** 1 Division of Nutritional Sciences, University of Illinois at Urbana-Champaign, Urbana, IL 61801 USA; 2 Department of Animal Sciences, University of Illinois at Urbana-Champaign, Urbana, IL 61801 USA; 3 Department of Nutrition and Exercise Physiology, University of Missouri, Columbia, MO 65211 USA; 4 Department of Biomedical Sciences, University of Missouri, Columbia, MO 65211 USA; 5 Department of Child Health, University of Missouri, Columbia, MO 65211 USA; 6 Dalton Cardiovascular Research Center, University of Missouri, Columbia, MO 65211 USA; 7 Department of Anesthesiology, University of Michigan, Ann Arbor, MI 48109 USA; 8 Department of Molecular & Integrative Physiology, University of Michigan, Ann Arbor, MI 48109 USA

## Abstract

Phytoestrogen-rich soy is known to ameliorate menopause-associated obesity and metabolic dysfunction for reasons that are unclear. The gut microbiota have been linked with the development of obesity and metabolic dysfunction. We aimed to determine the impact of soy on cardiometabolic health, adipose tissue inflammation, and the cecal microbiota in ovariectomized (OVX) rats bred for low-running capacity (LCR), a model that has been previously shown to mimic human menopause compared to sham-operated (SHM) intact control LCR rats. In this study, soy consumption, without affecting energy intake or physical activity, significantly improved insulin sensitivity and body composition of OVX rats bred for low-running capacity. Furthermore, soy significantly improved blood lipid profile, adipose tissue inflammation, and aortic stiffness of LCR rats. Compared to a soy-free control diet, soy significantly shifted the cecal microbial community of LCR rats, resulting in a lower Firmicutes:Bacteroidetes ratio. Correlations among metabolic parameters and cecal bacterial taxa identified in this study suggest that taxa *Prevotella*, *Dorea*, and *Phascolarctobacterium* may be taxa of interest. Our results suggest that dietary soy ameliorates adiposity, insulin sensitivity, adipose tissue inflammation, and arterial stiffness and exerts a beneficial shift in gut microbial communities in a rat model that mimics human menopause.

## Introduction

Approximately 50% of post-menopausal women seek alternative remedies for ovarian hormone loss-related symptoms due to the fear of potential adverse health effects that may come with chronic use of hormonal or non-hormonal medications^1, 2^. Lower prevalence of common menopausal-related vasomotor symptoms, including hot flashes and night sweats, in Japanese and Chinese compared to Caucasian women has drawn attention to soy consumptions^3^. Soy and soy products are widely used as alternative and complementary medicine therapies to alleviate menopausal related symptoms, and have been shown to reduce obesity and improve metabolic health in human and rodents^4–6^. Isoflavones are thought to be the active compounds in soy providing beneficial effects due to their structural similarity to endogenous estrogens produced by mammals. These phytoestrogens can bind to estrogen receptors (ER)-alpha and -beta and act as estrogen agonists or antagonists^7^. Unlike artificial endocrine disrupting compounds such as pesticides (e.g., dichloro-diphenyl-trichloroethane; DDT) and plasticizers (e.g., bisphenol A; BPA), phytoestrogens are generally viewed as natural compounds that exert health benefits, having anti-cancer, anti-atherosclerotic, and anti-osteoporotic properties^7, 8^.

Isoflavones usually exist in food as biological inactive forms, such as genistin and daizin, which are present in soy as β-D-glycosides. These glycosides can be hydrolyzed and deconjugated by β-glycosidases synthesized by the intestinal microbiota to form biologically active aglycone forms of isoflavones, such as genistein and daidzein. The aglycone isoflavone forms can then be absorbed and further metabolized into equol. S-(-)equol, the natural diastereoisomer of equol, is exclusively produced by equol-producing bacteria in the intestine of humans and rodents and has a higher estrogenic potency than other isoflavone forms. Although still unclear, the ability of producing equol has been hypothesized to be crucial for obtaining the health benefits from a soy-rich diet^9^, suggesting a critical role of intestinal microbes on isoflavone metabolism.

The mechanisms by which soy may ameliorate menopause-associated obesity and metabolic dysfunction are unclear. The gut microbiota has been shown to mediate gut integrity and impact systemic inflammation associated with obesity. For instance, lower Firmicutes:Bacteroidetes ratio of gut microbiota has been associated with a lean phenotype^10^. However, the association between the gut microbiota, soy consumption, and menopause- associated obesity has not been established. A previous study demonstrated that male Sprague-Dawley rats fed a high-cholesterol diet with added soy-milk had a 2.3-fold increase in the relative abundance of *Lactobacillus spp*. compared to those fed the same high-cholesterol diet without soy supplementation^11^. Certain *Lactobacillus spp*., such as *L*. *mucosae* ^12^ and *L*. *Niu-O16* ^13^ are known equol producers that can convert daidzein into equol *in vitro*. Therefore, changes in the gut microbial communities may represent one potential mechanism for soy’s beneficial metabolic impacts on those with ovarian hormone insufficiency.

Herein, we aimed to determine the impact of soy on insulin sensitivity, adiposity, adipose tissue inflammation, vascular function, and the cecal microbiota of ovariectomized rats bred for low-running capacity, a model that has been previously shown to mimic human menopause by our research group^14^. We hypothesized that soy consumption would improve whole body insulin sensitivity and vascular health, hinder fat mass gain and adipose tissue inflammation, and beneficially impact the gut microbial community by increasing the relative abundance of known equol-producing bacteria such as *Lactobacillus*, *Enterococcus*, and *Adlercreutzia* and decreasing the Firmicutes:Bacteroidetes ratio.

## Results

### Diet analysis

Forty 27-wk-old LCR female rats were either ovariectomized (OVX) or sham operated (SHM) and *ad libitum* fed either soy-rich (soy) or soy-free (control) diets for 28 wk in a 2 × 2 factorial arrangement (n = 10/group): (1) OVX/soy; (2) SHM/soy; (3) OVX/control; (4) SHM/control. Diets utilized in the current study were isocaloric and comparable in protein, fat, digestible carbohydrate (i.e., nitrogen free extract), and total dietary fiber concentrations (Table 1). Phytoestrogen was not detected in the control, soy-free diet, whereas soy-rich diet contained 585 mg/kg diet of total phytoestrogen.

**Table 1:** Ingredient and analyzed chemical composition of the diets.

*Ingredient*	Control	Soy
	g/kg diet (as-fed basis)	
Corn gluten meal (60% protein)	188	0
Soybean meal (48% protein)	0	260
Corn	388	358
Wheat, soft	231	230
Wheat middlings	73.0	46.0
DL-methionine. FG (99%)	1.0	1.0
L-lysine HCl, FG (78%)	8.0	1.0
Soybean oil	16.0	20.0
Cellulose	58.9	50.6
Mineral mix	5.0	5.0
Calcium phosphate	10.0	8.0
Calcium carbonate	13.0	13.0
Sodium chloride, iodized	2.5	2.5
Magnesium oxide, FG (58%)	0.5	0.5
Vitamin mix	4.0	4.0
Choline chloride, FG (60%)	1.6	0.4
Analyzed chemical composition		
Dry matter (DM, **%)**	85.2	90.7
	–DM basis–	
Organic matter (%)	95.6	94.2
Ash (%)	4.4	5.8
Crude protein (%)	22.2	20.8
Acid hydrolyzed fat (%)	6.6	6.0
Nitrogen Free Extract (%)	47.6	47.3
Total dietary fiber (%)	19.2	20.1
Insoluble dietary fiber (%)	16.8	19.3
Soluble dietary fiber (%)	2.5	0.8
Oligosaccharides (%)		
Raffinose (%)	0	0.4
Stachyose (%)	0	1.3
Verbascose (%)	0	0.1
Gross energy (kcal/g DM)	4.7	4.5
Calculated metabolizable energy(Atwater factor, kcal/g DM)		
	3.4	3.3
Calculated metabolizable energy (modified Atwater factor, kcal/g DM)		
	3.0	2.9
Total phytoestrogen (mg/kg)	0	585
Daidzin/daidzein (mg/kg)	0	290
Genistin/genistein (mg/kg)	0	190
Glycitin/glycitein (mg/kg)	0	105

### Soy consumption lowered weight gain and adiposity and increased cecal weight without affecting food intake

**Figure 1:**
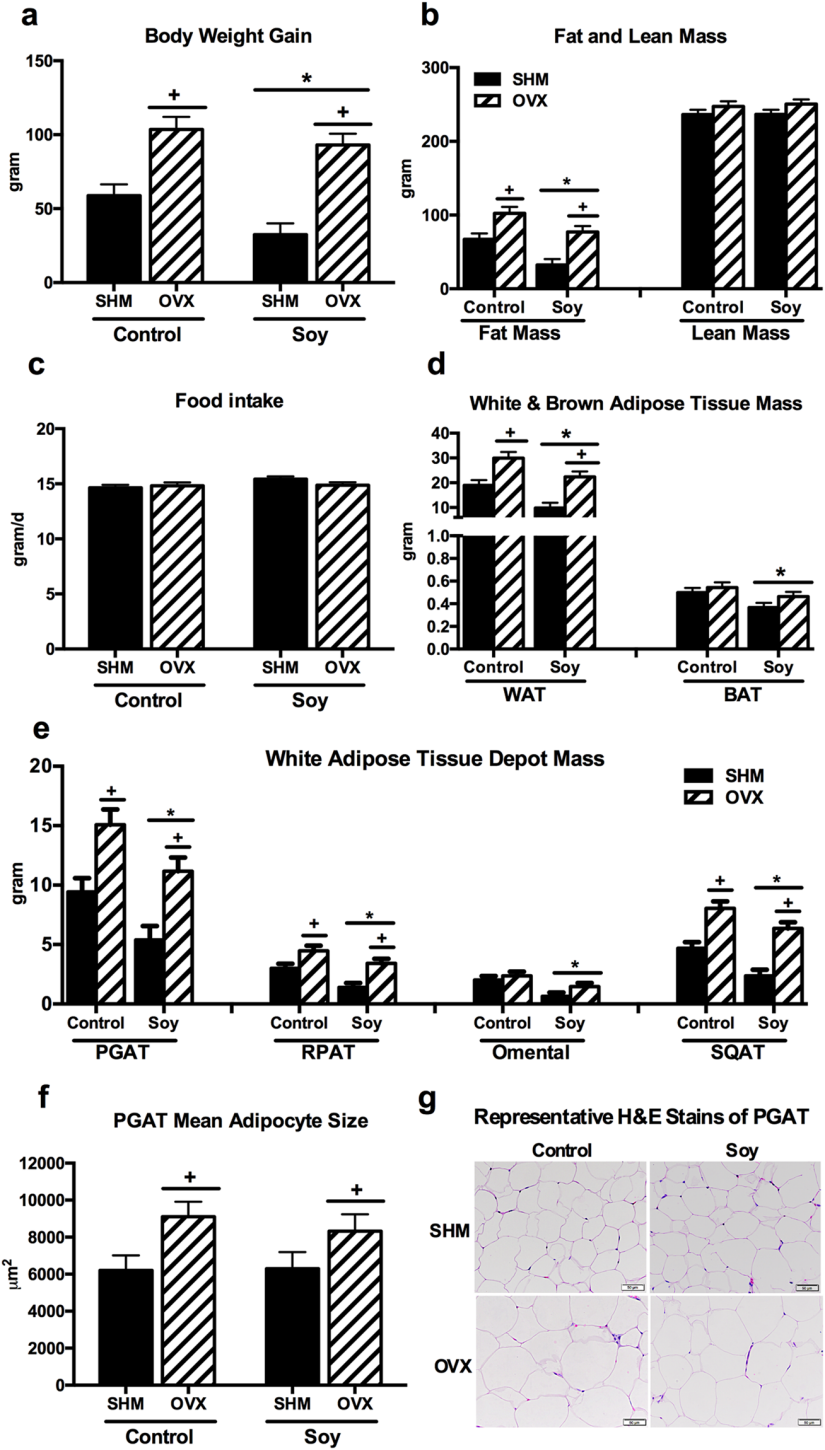
Animal characteristics. OVX led to greater body weight gain (**a**), fat (**b**), and white adipose tissue mass (**d**), while rats fed soy diet gained less body weight, fat, and white adipose tissue mass. Food intake (**c**) was not affected by the treatment, white and brown adipose tissue mass (**d**), white adipose tissue depot mass (**e**), perigonadal adipose tissue (PGAT) mean adipocyte size (**f**), and representative H&E stains of PGAT (**g**) of ovariectomized (OVX) vs. sham-operated (SHM) rats bred for low-running capacity fed either a soy-rich or soy-free diet. Data were analyzed using the Mixed Models procedure of SAS 9.3 with diet and surgery being fixed effects and animal ID being a random effect. When a main effect was significant, post hoc pairwise comparisons were performed using Tukey’s multiple comparison tests. Data are least-squares means ± SEM, n = 4–10. ^+^Denotes main surgery effect (OVX vs. SHM); *Denotes main diet effect (Control vs. Soy), *P* < 0.05.

The initial mean BW of rats was not different among treatment groups (data not shown), but OVX rats had a greater (*P* < 0.05) final BW when compared to SHM rats after 28 wk of feeding (Table 2). Throughout the study, OVX rats gained more (*P* < 0.05) weight than SHM rats and soy-fed rats gained less (*P* < 0.05) weight than controls (Fig. 1a). Similarly, OVX rats gained more (*P* < 0.05) fat mass than SHM rats. Soy-fed rats gained less (*P* < 0.05) fat mass than controls, whereas lean mass was not different among treatment groups (Fig. 1b). Daily average food intake did not differ among treatment groups prior to sacrifice (Fig. 1c). Soy-fed rats had greater (*P* < 0.05) total cecum weight, including cecal tissue and contents, than controls (4.72 ± 0.14 vs. 3.76 ± 0.15). Total white adipose tissue weight (sum of PGAT, RPAT, omental, and SQAT fat pads) was higher (*P* < 0.05) in the OVX than SHM groups, but soy-fed rats had lower (*P* < 0.05) total white adipose tissue weight compared to controls (Fig. 1d). BAT weight was lower (*P* < 0.05) in rats fed soy than those fed the control diet (Fig. 1d). In terms of white adipose tissue distribution, visceral (PGAT and RPAT) and SQAT fat pads were greater (*P* < 0.05) in the OVX rats than SHM, whereas soy reduced (*P* < 0.05) all fat pad weights (Fig. 1e), including the omental depot (Fig. 1e). Greater (*P* < 0.05) adipocyte hypertrophy was observed with OVX, but was not affected by diet (Fig. 1f,g).

**Table 2:** Body weight and fasting blood parameters of ovariectomized (OVX) vs. sham-operated (SHM) rats bred for low-running capacity fed either a soy-rich (Soy) or soy-free diet (Control).

	Control^2^		Soy		*p* values		
	SHM	OVX	SHM	OVX	Diet	Surgery	Diet*Surgery
Final body weight (g)	313 ± 11.7	366 ± 13.1	288 ± 11.7	347 ± 11.7	0.07	<0.01	0.77
Fasting insulin (μU/mL)	35.2 ± 11.5	61.9 ± 12.2	28.1 ± 10.9	54.8 ± 10.9	0.68	0.01	0.27
Fasting glucose (mg/dL)	190 ± 18.3	217 ± 20.5	175 ± 18.3	182 ± 18.3	0.20	0.37	0.59
Fasting triglycerides (mg/dL)	126 ± 15.0	111 ± 16.7	90.2 ± 15.0	123 ± 15.0	0.43	0.58	0.13
Fasting cholesterol (mg/dL)	123 ± 6.68	119 ± 7.47	95.0 ± 6.68	111 ± 6.68	0.02	0.39	0.14
Fasting HDL-c (mg/dL)	40.5 ± 1.95	36.5 ± 2.17	34.3 ± 1.95	37.2 ± 1.95	0.18	0.79	0.09
Fasting LDL-c (mg/dL)	5.60 ± 0.80	7.13 ± 0.90	4.30 ± 0.80	6.80 ± 0.80	0.10	<0.01	0.50
Fasting NEFA (mmol/L)	0.69 ± 0.07	0.53 ± 0.08	0.56 ± 0.07	0.55 ± 0.07	0.46	0.23	0.31

Values are least-squared means ± SEM, n = 8–10/group. Means in a row without a common superscript letter differ, *P* < 0.05.

### Liver triglyceride content and fasting blood parameters

**Figure 2:**
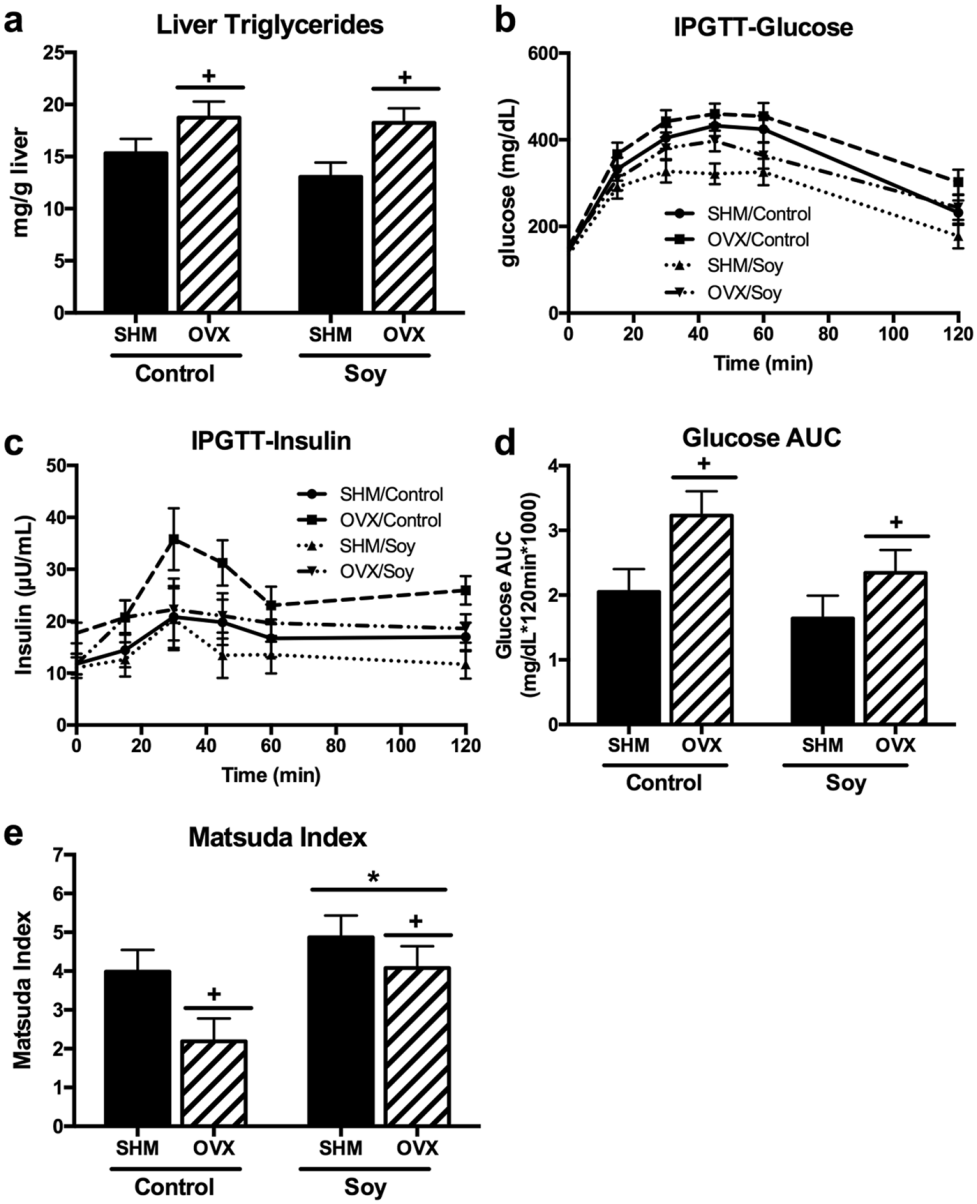
OVX led to greater concentrations of liver triglycerides (**a**). Intraperitoneal glucose tolerance test was performed at 19wk post-surgery and glucose (**b**) and insulin (**c**) changes over time was plotted. Corresponding glucose area under the curve (AUC) (**d**) and Matsuda Index (**e**) was calculated from ovariectomized (OVX) vs. sham-operated (SHM) rats bred for low-running capacity fed either a soy-rich or soy-free diet. Data were analyzed using the Mixed Models procedure of SAS 9.3 with diet and surgery being fixed effects and animal ID being a random effect. When a main effect was significant, post hoc pairwise comparisons were performed using Tukey’s multiple comparison tests. Data are least-squares means ± SEM, n = 4–10. ^+^Denotes main surgery effect (OVX vs. SHM); *Denotes main diet effect (Control vs. Soy), *P* < 0.05.

OVX had elevated (*P* < 0.05) liver triglyceride concentrations compared to SHM rats and soy consumption did not significantly lower liver triglyceride content in either group (Fig. 2a). Fasting blood parameters measured are shown in Table 2. OVX led to 85% greater (*P* < 0.05) fasting insulin concentrations compared to SHM rats. Fasting glucose and triglyceride concentrations were not affected by diet or surgery. Glucose did tend to increase following OVX and decrease following soy consumption, but these differences did not approach statistical significance (*P* = 0.2 and *P* = 0.37, respectively). Soy-fed rats had lower (*P* < 0.05) fasting cholesterol concentrations compared to controls. OVX rats had greater (*P* < 0.05) fasting LDL-c concentrations than SHM rats, whereas HDL-c concentrations were not affected by diet or surgery interventions. Fasting serum NEFA did not differ among treatment groups.

### Soy enhances whole body insulin sensitivity

Glucose and insulin concentrations in response to the IPGTT challenge are shown in Fig. 2b and c. IPGTT glucose area under the curve (AUC) revealed that OVX rats were less (*P* < 0.05) glucose tolerant than SHM rats (Fig. 2d). The Matsuda Index of insulin sensitivity, calculated utilizing glucose and insulin concentrations obtained from IPGTT, confirmed that OVX rats had lower (*P* < 0.05) insulin sensitivity than SHM; further, soy-fed OVX rats experienced an improvement in OVX-associated in insulin sensitivity (i.e., Matsuda Index greater than >2.5) (Fig. 2e). Moreover, the Matsuda Index revealed that soy-fed rats in both SHM and OVX groups had greater (*P* < 0.05) insulin sensitivity compared to controls.

### Ovariectomy reduces energy expenditure and spontaneous physical activity

**Figure 3:**
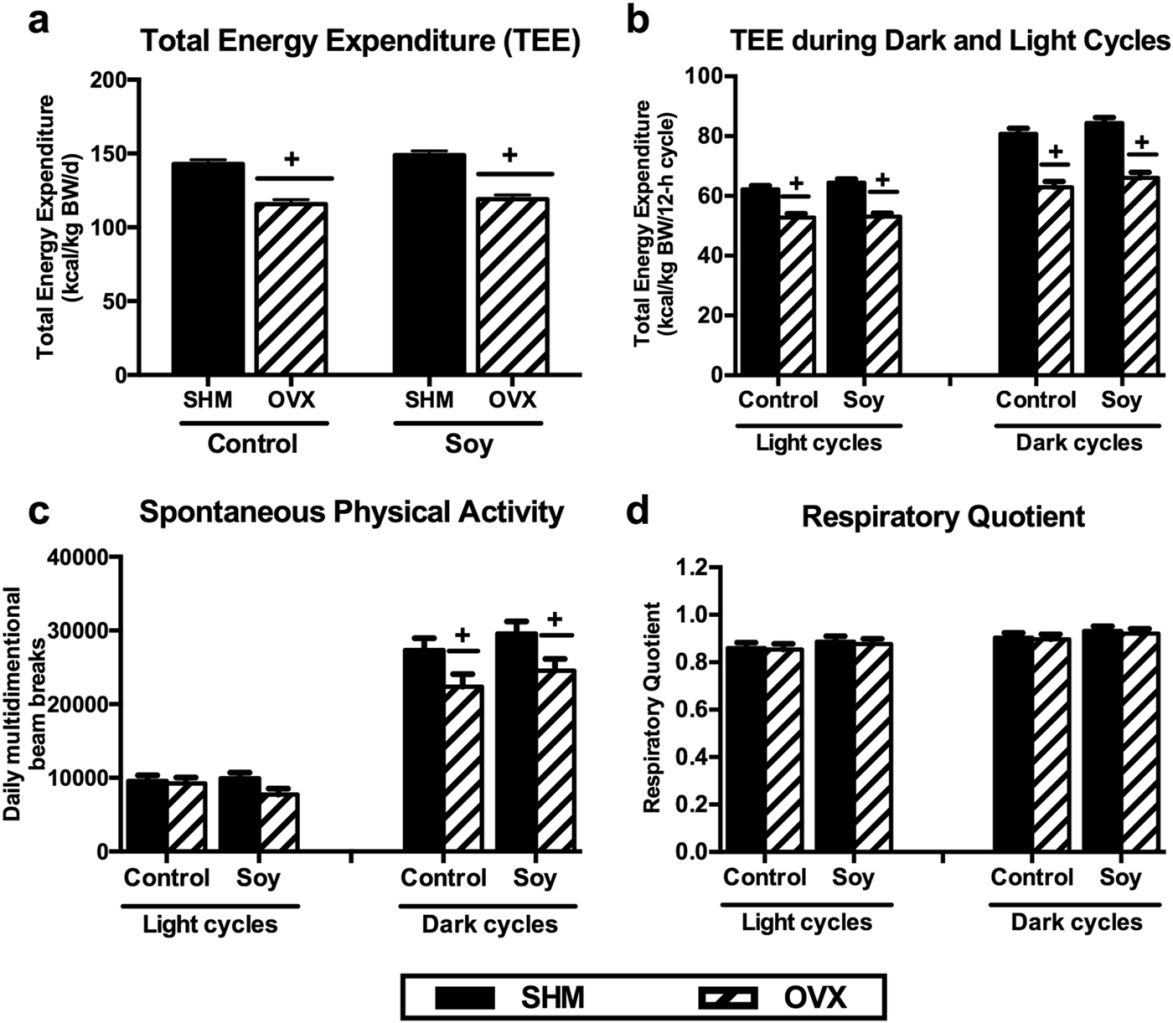
Total energy expenditure (TEE) (**a**), TEE during dark and light cycles (**b**), spontaneous physical activity (SPA) (**c**), and respiratory quotient (RQ) (**d**) of ovariectomized (OVX) vs. sham-operated (SHM) rats bred for low-running capacity fed either a soy-rich or soy-free diet. Data were analyzed using the Mixed Models procedure of SAS 9.3 with diet and surgery being fixed effects and animal ID being a random effect. When a main effect was significant, post hoc pairwise comparisons were performed using Tukey’s multiple comparison tests. Data are least-squares means ± SEM, n = 8–10. ^+^Denotes main surgery effect (OVX vs. SHM); *Denotes main diet effect (Control vs. Soy), *P* < 0.05.

OVX reduced (*P* < 0.05) total energy expenditure (TEE) (Fig. 3a), which was likely attributed to a decrease during the dark (i.e., active) cycle (Fig. 3b). Similarly, spontaneous physical activity (SPA) was reduced (*P* < 0.05) by OVX during the dark cycle, it was not significantly affected by soy consumption (Fig. 3c). Respiratory quotient (RQ) did not differ among groups (Fig. 3d), suggesting that substrate utilization was not significantly affected by diet or OVX.

### Ovariectomy increases, and soy decreases, adipose tissue inflammatory mRNA expression

**Figure 4:**
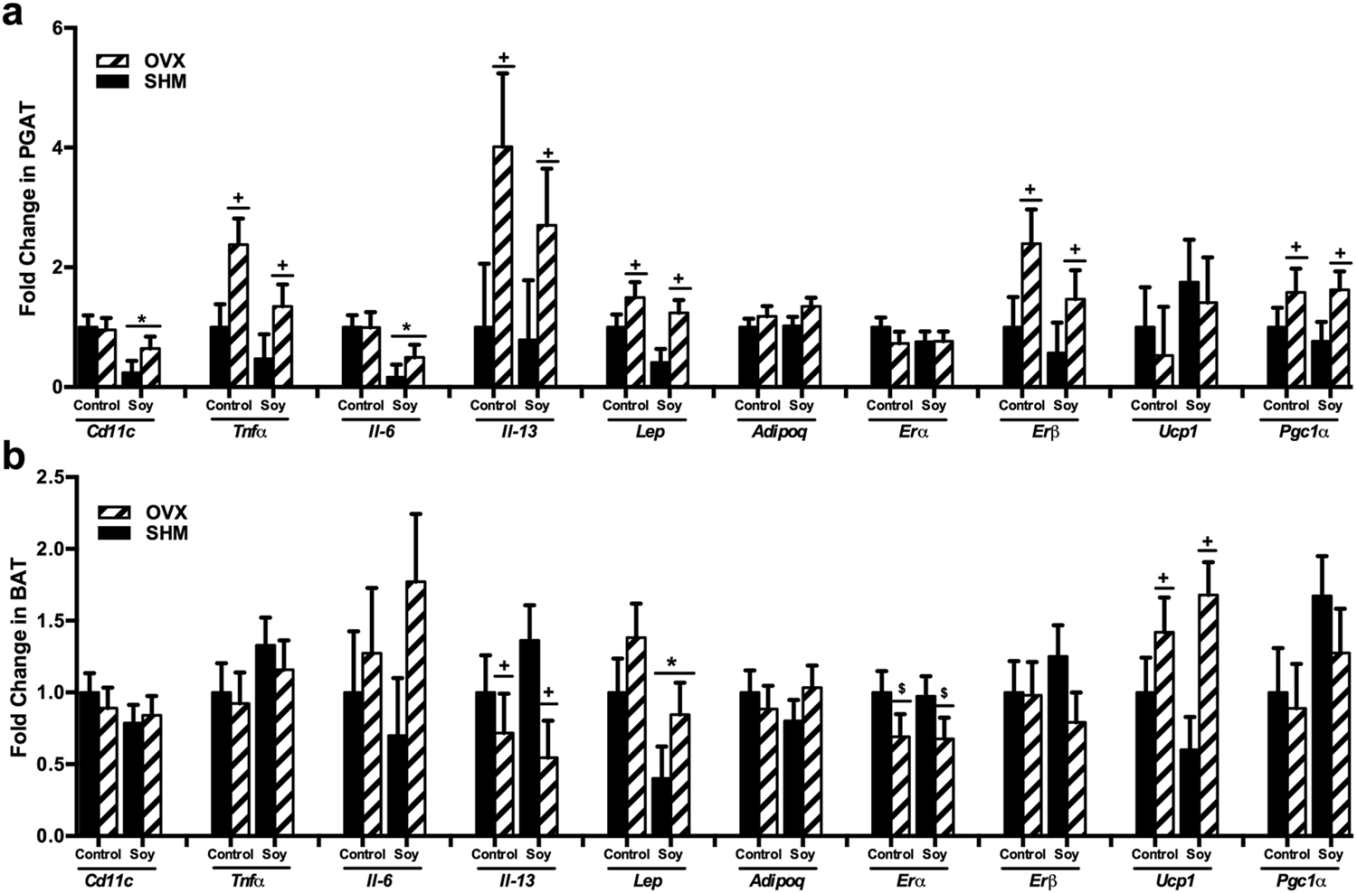
Gene expression of perigonadal (PGAT) (**a**) and brown (**b**) adipose tissue of ovariectomized (OVX) vs. sham-operated (SHM) rats bred for low-running capacity fed either a soy-rich or soy-free diet. Data were analyzed using the Mixed Models procedure of SAS 9.3 with diet and surgery being fixed effects and animal ID being a random effect. When a main effect was significant, post hoc pairwise comparisons were performed using Tukey’s multiple comparison tests. Data are least-squares means ± SEM, n = 8–10. ^+^Denotes main surgery effect (OVX vs. SHM) *P* < 0.05; ^$^Denotes trends in surgery effect, *P* < 0.1; *Denotes main diet effect (Control vs. Soy), *P* < 0.05.

Soy-fed rats had lower (*P* < 0.05) mRNA expression of the inflammatory macrophage marker, CD11c, and the proinflammatory cytokine, IL-6 in PGAT (Fig. 4a). In BAT, soy reduced (*P* < 0.05) expression of leptin, the adipokine classically produced and secreted from white adipose tissue and indicative of “whitening” of BAT (Fig. 4b). OVX increased (*P* < 0.05) the mRNA expression of the classic proinflammatory cytokine, TNFα, as well as a cytokine associated with alternative macrophage activity, IL-13, and leptin in PGAT. In BAT, OVX increased (*P* < 0.05) expression of UCP1 and lowered (*P* < 0.05) expression of IL-13.

### Soy decreases aortic stiffness but does not affect endothelium-dependent and independent relaxation responses

**Figure 5:**
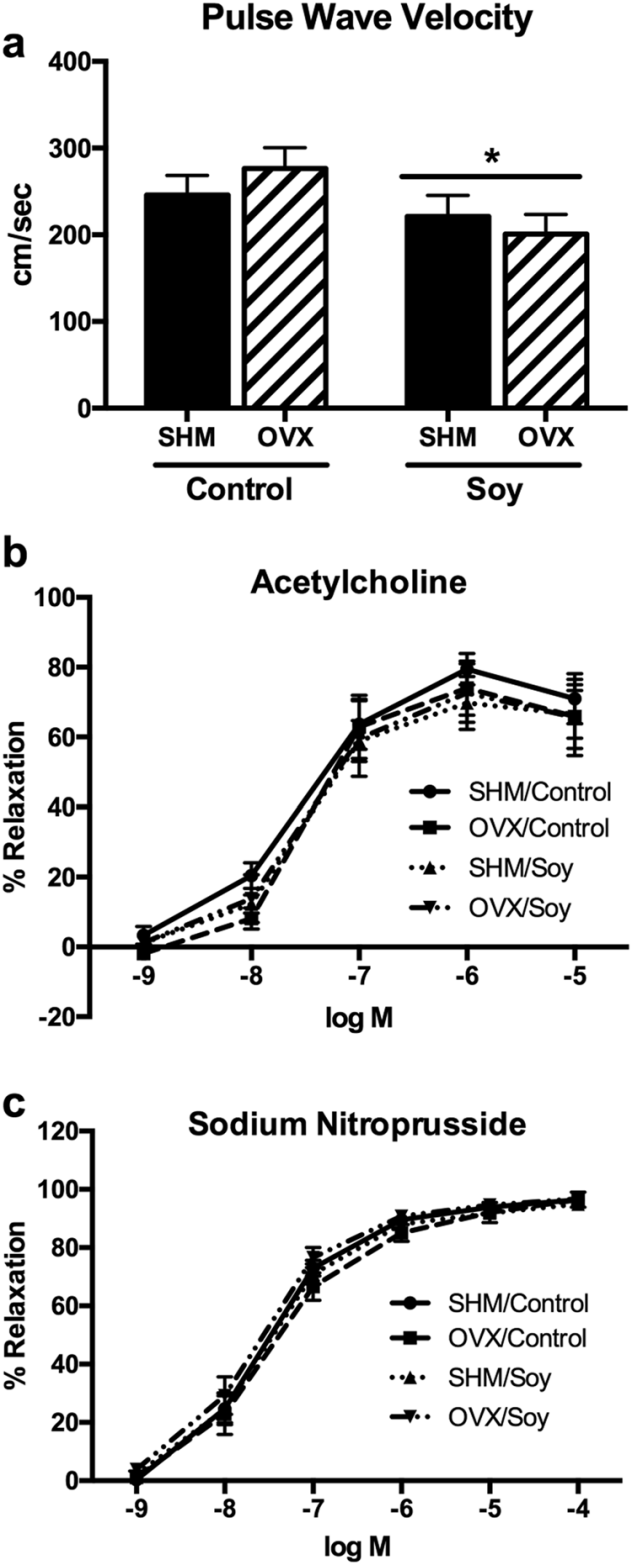
Aortic stiffness and vasomotor function in ovariectomized (OVX) vs. sham-operated (SHM) rats bred for low-running capacity fed either a soy-rich or soy-free diet. (**a**) aortic pulse wave velocity; (**b**) acetylcholine-induced relaxation in aortic rings; (**c**) sodium nitroprusside-induced relaxation in aortic rings. Data were analyzed using the Mixed Models procedure of SAS 9.3 with diet and surgery being fixed effects and animal ID being a random effect. When a main effect was significant, post hoc pairwise comparisons were performed using Tukey’s multiple comparison tests. Data are least-squares means ± SEM, n = 8–10. ^+^Denotes main surgery effect (OVX vs. SHM); *Denotes main diet effect (Control vs. Soy), *P* < 0.05.

Aortic pulse wave velocity (PWV), a marker of arterial stiffness, was lowered (*P* < 0.05) with soy feeding (*P* < 0.05) but not significantly affected by OVX (Fig. 5a). On the other hand, neither acetylcholine or sodium nitroprusside-mediated aortic relaxation responses were affected by OVX or soy treatment (Fig. 5b,c).

### Diet robustly impacts the composition of the cecal microbiota

**Figure 6:**
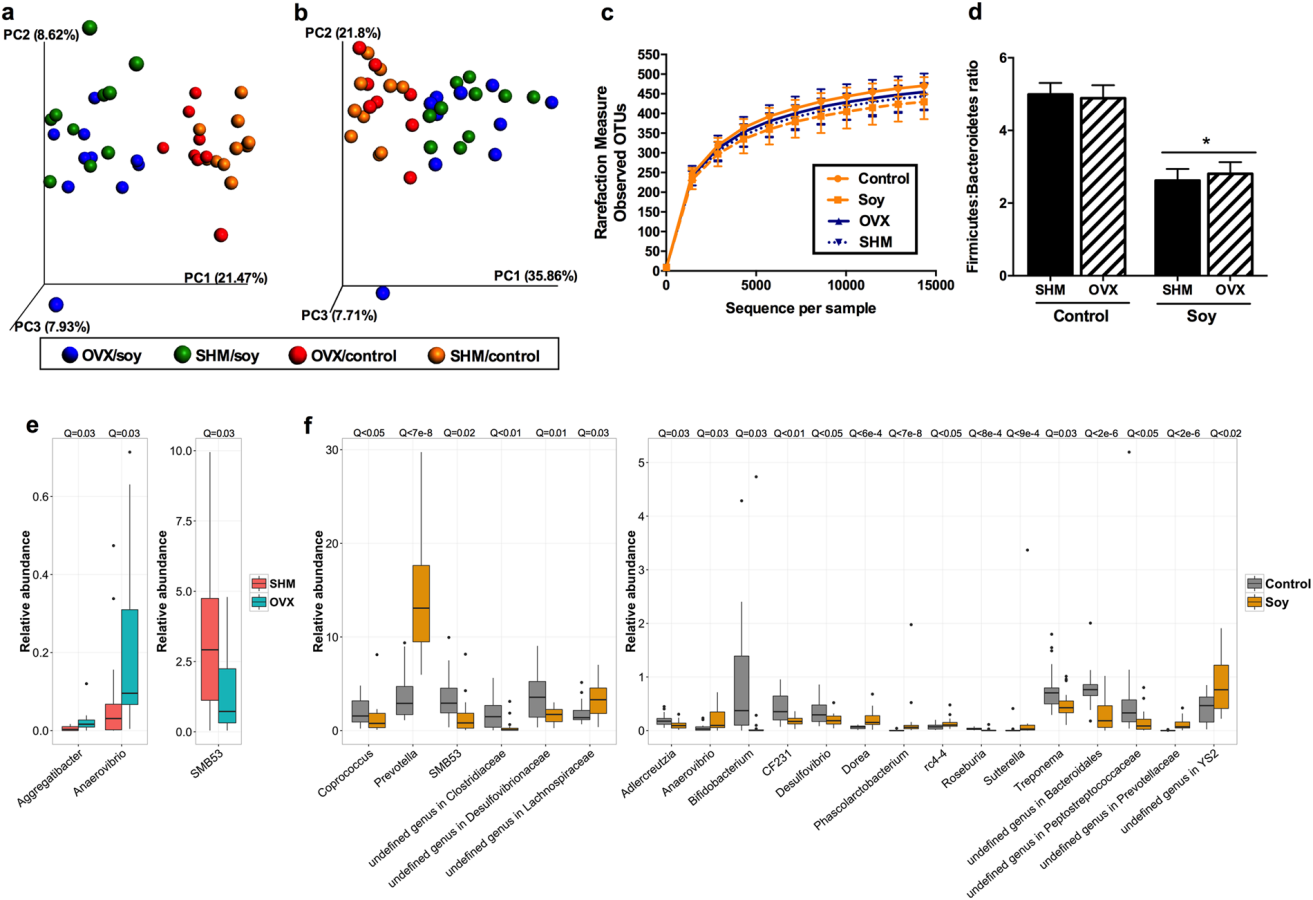
Cecal microbial communities of ovariectomized (OVX) vs. sham-operated (SHM) rats bred for low-running capacity fed either a soy-rich or soy-free diet. Principal coordinates analysis plots of unweighted (**a**) and weighted (**b**) UniFrac distances of cecal microbial communities performed on the 97% OTU abundance matrix revealed a distinct separation (*P* = 0.01) between rats fed control vs. soy diets, but did not differ between OVX and SHM groups. Alpha diversity measures (**c**) suggest lower (*P* < 0.05) species richness in rats fed soy than those fed control diet. Firmicutes:Bacteroidetes ratio (**d**) were lower (*P* < 0.05) in rats fed soy than those fed control diet. Relative abundances of the differentially abundant taxa (*P* < 0.05) between OVX vs. SHM (**e**) and Soy vs. Control diets (**f**). False discovery rate corrected P values (Q vales) were calculated using MaAsLin. The box represents the first and third quartiles (i.e., the 25^th^ and 75^th^ percentiles); error bars indicate 95% confidence interval of median.

Principal coordinates analysis of unweighted (Fig. 6a) and weighted (Fig. 6b) UniFrac distances performed on the 97% OTU abundance matrix of cecal microbiota revealed a distinct separation (*P* = 0.01) between rats fed control and soy diets. Beta diversity did not differ between OVX and SHM groups. Alpha diversity measures suggested that the soy diet led to lower (*P* < 0.05) species richness than the control diet [observed OTUs at the 97% level: 430 ± 44.1 compared with 471 ± 22.0 (soy diet compared with control diet, *P* < 0.01); Chao 1 index: 476 ± 43.9 compared with 519.7 ± 18.9 (*P* < 0.01); phylogenetic diversity whole tree matrix: 29.3 ± 1.89 compared with 30.7 ± 1.23 (*P* < 0.05)] (Fig. 6c). Similar to beta-diversity, species richness did not differ between OVX and SHM groups.

### Microbial taxonomic shifts due to ovariectomy and soy-rich diet

Greengenes classifier assigned usable raw reads to 11 phyla, 35 families, and 55 genera. The most abundant phyla included Firmicutes (66.9% of sequences), Bacteroidetes (20.3% of sequences), and Proteobacteria (10.3% of sequences). Rats fed a soy-rich diet had a lower (*P* < 0.05) relative abundance of Firmicutes, but higher (*P* < 0.05) relative abundance of Bacteroidetes (Supplementary Table S1), resulting in a lower (*P* < 0.001) Firmicutes: Bacteroidetes ratio compared to those fed control diet (Fig. 6d). Firmicutes: Bacteroidetes ratio between OVX and SHM rats was not different.

We utilized MaAsLin analysis to assess the specific taxonomic changes due to soy consumption and the loss of ovarian hormone production. OVX led to an increase (*P* < 0.05) in relative abundance of *Aggregatibacter* and *Anaerovibrio*, but a decrease in the relative abundance of SMB53 (Fig. 6e). Soy consumption was linked to a greater (*P* < 0.05) relative abundance of multiple genera, including *Prevotella*, an undefined genus in family *Lachnospiraceae*, *Dorea*, *Phascolarctobacterium*, *rc4-4*, *Sutterella*, an undefined genus in family *Prevotellaceae*, and an undefined genus in order *YS-2* (Fig. 6f). On the other hand, soy consumption was linked to a lower (*P* < 0.05) relative abundance of *Coprococcus*, *SMB53*, an undefined genus in family *Clostridiaceae*, an undefined genus in family *Desulfovibrionaceae*, *Adlercreutzia*, *Bifidobacterium*, *CF231*, *Desulfovibrio*, *Roseburia*, *Treponema*, an undefined genus in order *Bacteroidales*, and an undefined genus in family *Peptostreptococcaceae*.

### Significant correlations identified among cecal microbial taxa and physiological parameters and gene expression

**Figure 7:**
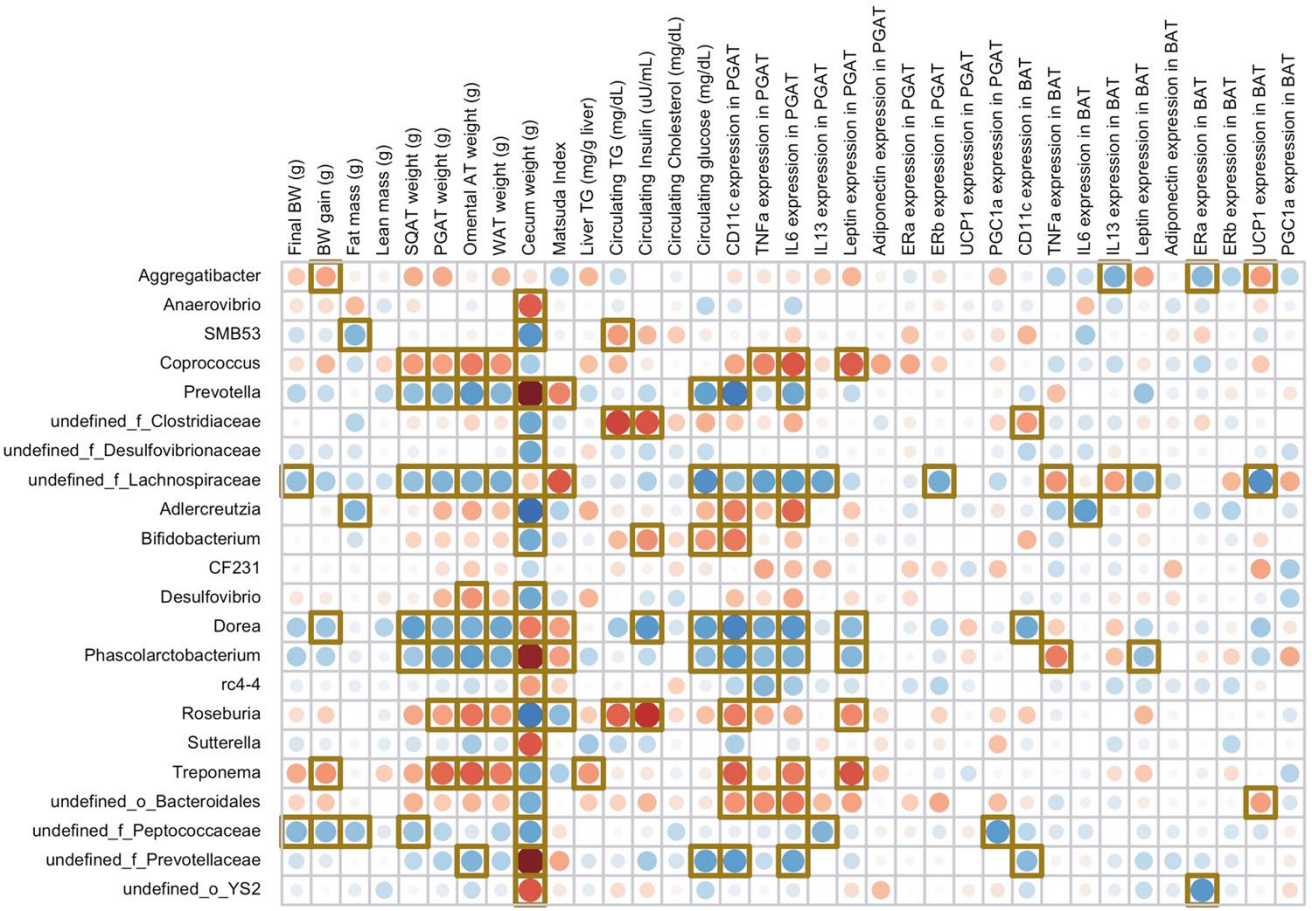
Correlations among differentially abundant taxa within the cecal microbial community identified through MaAsLin analysis and physiological parameters and gene expression data. Red dots represent positive correlations whereas blue dots represent negative correlations; yellow square box denotes statistical significance (*p* < 0.05) observed using Spearman correlation.

Correlations among differentially abundant taxa within the cecal microbial community identified through MaAsLin analysis and physiological parameters and gene expression data were identified (Fig. 7). The relative abundance of *Prevotella*, *Dorea*, and *Phascolarctobacterium*, all taxa that increased (*P* < 0.05) with soy supplementation, were negatively (*P* < 0.05) correlated with SQAT, PGAT, omental AT, and total white AT weights, circulating glucose concentration, and CD11c and IL6 expression in PGAT. These taxa were positively (*P* < 0.05) correlated with cecum weight and Matsuda Index. The relative abundance of *Dorea* and *Phascolarctobacterium* were also negatively (*P* < 0.05) correlated with TNFα expression in PGAT. Cecum weight, which was greater (*P* < 0.05) with soy supplementation, was highly associated (*P* < 0.05) with changes in cecal microbial taxa.

## Discussion

Menopause, part of the natural aging process in women characterized by cessation of ovarian hormone production, is associated with increased adiposity, insulin resistance, dyslipidemia, and inflammation, contributing to an elevated risk of metabolic diseases, including cardiovascular disease (CVD) and type 2 diabetes^15^. This metabolic profile is recapitulated in OVX rodents, making them a suitable animal model of human menopause. OVX not only leads to increased total adiposity, but also increases adipose tissue inflammation, which we previously found to precede the development of systemic insulin resistance^14^. Given the strong relationship between adipose tissue inflammation and systemic metabolic dysfunction, determining safe and efficacious approaches to reduce adipose tissue inflammation in settings of ovarian hormone loss is a vital area of investigation. Soy is commonly used as a natural remedy for menopause-related vasomotor symptoms due to its high isoflavone (namely phytoestrogen) content. Whereas many of the adverse metabolic effects of menopause are due to loss of estrogen (i.e., 17 β-estradiol) in particular, phytoestrogens are selective estrogen receptor modulators (SERMs) which bind estrogen receptors, albeit with much lower affinity than 17 β-estradiol. Randomized controlled trials have demonstrated many cardiometabolic protective effects of soy, including improvements in insulin sensitivity and blood lipids^16^, as well as direct vascular improvements^17, 18^. Of particular importance, a comprehensive review of 75 interventions aimed to identify dietary factors to reduce CVD risk found that soy was one of only two factors that reduced arterial stiffness^19^. Unfortunately, the mechanisms by which soy benefits metabolic function remain unclear. Interestingly, there is increasing evidence that dietary soy may improve adipose tissue metabolism. A recent study in OVX rats showed that soy reduced markers of inflammation and oxidative stress in adipose tissue^20^. Other reports have shown that the soy isoflavone, genistein reduced adipocyte size and increased adipose tissue fat oxidation following OVX^21^; similar findings were reported for another phytoestrogen, daidzein^22^, which also was shown to reduce adipocyte inflammation in a cell culture model^23^.

Since the gut microbiota are known to be essential in transforming the biologically inactive forms of isoflavones present in food into active metabolites that have higher estrogenic potency, determining associations between soy consumption, menopause-related metabolic dysfunction, and the gut microbiota is an important area of study. Herein, we demonstrated in a low aerobically fit rodent model of human menopause that a diet high in phytoestrogen-rich soy: (1) improved body composition, blood lipid profile, systemic insulin sensitivity, and arterial stiffness; (2) lowered inflammatory gene expression in visceral adipose tissue, and leptin in BAT; (3) shifted the cecal microbial community resulting in lowered Firmicutes:Bacteroidetes ratio that is associated with a lean phenotype. To translate the dietary intervention used here to humans, the phytoestrogen dose in the soy diet corresponds to ~9 mg/day (~30 mg/kg BW). In adult humans in the United States, estimates indicate that average dietary soy isoflavone consumption ranges from 0.17–6.31 mg/day (~0.002–0.09 mg/kg BW)^24^ using an estimated rodent to human dose conversion from Reagan-Shaw *et al*.^25^. Thus, the experimental dose in this study would translate to ~2.4 mg/kg in humans, a dose that would likely require isoflavone supplementation above normal dietary intake.

Similar to the current study, previous studies have demonstrated anti-obesity effects of soy in obese rodents^4, 5^. However, evidence of weight loss due to long-term soy supplementation is limited in obese humans^26^, potentially due to the confounding factors of hypocaloric diet and physical activity interventions in most human trials. A recent clinical trial demonstrated that a soy-based meal replacement led to comparable weight loss compared to a lifestyle intervention group (i.e., dietary and physical activity modifications) in obese men and women^6^. Two groups of overweight or obese subjects received either dietary counseling and fitness instruction (lifestyle intervention group) or received a low-calorie meal-replacement high in soy protein (meal replacement group) for six months. In that study, the meal replacement group had a pronounced reduction in adipokines fetuin A and resistin compared to the lifestyle group, reinforcing the potential anti-obesity effect of soy in humans.

Beneficial effects of soy are commonly attributed to its high phytoestrogen concentration, including genistein, daidzein, and glycitein. Kim *et al*.^27^ demonstrated that feeding 1500 mg/kg of genistein, but not a lower dose of 150 mg/kg, for 21 d significantly reduced adiposity in OVX mice. The phytoestrogen-rich soy diet fed in the current study only provided 190 mg/kg of genistin/genistein, but was sufficient to reduce adiposity, suggesting that other components of the soybean matrix such as soyasaponins, phospholipids, other forms of isoflavones (e.g., daidzin/daidzein and glycitin/glycitein), or oligosaccharides may also have positive effects on fat reduction. Indeed, another clinical trial demonstrated that whole soy, but not daidzein alone, improved cardiovascular risk in postmenopausal women^28^, supporting the notion that the food matrix may play a role in the health benefits of soy. Moreover, Kurrat *et al*.^29^ reported that life-long, but not short-term, exposure to isoflavones protected Wistar rats from obesity after ovariectomy. Therefore, the weight loss benefits observed in the current study may be partly due to the length of dietary intervention. Although the aforementioned animal studies have shown promising results, the effects of soy or individual phytoestrogens on body composition in post-menopausal women are not conclusive^30, 31^.

Similar to previous findings in humans^32^, we demonstrated that soy decreased serum cholesterol concentrations in OVX rats. Menopause also associates with elevated circulating and liver triglycerides (i.e., hepatic steatosis), increasing risks of CVD^33, 34^. Previous studies using obese mice showed that daidzein reduced hepatic steatosis, but this effect only existed at a high dose of 500 mg/kg diet^35^. In the current study, soy provided ~290 mg of daidzin/daidzein/kg diet and did not significantly reduce circulating or liver triglycerides, but did reduce aortic PWV, a measure of aortic stiffness. Interestingly, a decrease in aortic stiffness using PWV was also recently demonstrated in humans when supplemented with 80 mg soy isoflavones/d^36^. Notably, in humans, aortic stiffness is an independent predictor of cardiovascular disease and all-cause mortality^37–39^. Adipocyte hypertrophy is associated with cell death and adipose tissue macrophage infiltration, which leads to adipose tissue inflammation and affects obesity-associated adipose tissue remodeling^40^. In the current study, OVX led to adipocyte hypertrophy and elevated expression of TNFα in PGAT, while soy reduced mRNA expression of markers of adipose tissue inflammatory macrophage infiltration (CD11c) and inflammation (IL-6). Although the mechanism could not be determined in this study, it is noteworthy that the soy peptide Phe-Leu-Val has been shown to reduce insulin resistance and TNFα-induced inflammation *in vitro* ^41^. Daidzein has also been shown to decrease proinflammatory TNFα mRNA expression in mouse adipose tissue^35^. However, in humans, the effects of lowering inflammation through long-term soy supplementation are inconclusive^42–44^. It should be noted that only gene expression was measured in the present study, and the up-regulated inflammatory genes were not validated with protein measures. Although we have previously shown strong correlations between WAT inflammatory gene and protein expression^45^, future studies should measure inflammatory proteins to validate the inflammatory changes observed here.

Whereas all rodent guts contain equol-producing bacteria, only 30–50% of humans harbor equol-producing bacteria^46, 47^, which may explain differential responses regarding phytoestrogen consumption between rodents and humans. S-(-)equol is an exclusively microbially-derived metabolite converted from daidzein, with selective affinity for ERβ. The conversion from daidzein to equol is thought to be important for obtaining beneficial outcomes from ingesting soy^9^, highlighting the importance of investigating soy-mediated changes in gut microbial communities. In the current study, the gut microbiota of soy-fed rats clustered separately from those fed the soy-free control diet. Rats fed soy had a lower alpha diversity and Firmicutes:Bacteroidetes ratio. An increase in Firmicutes:Bacteroidetes ratio has been associated with obesity and adiposity^48^. In both SHM and OVX rats, a soy-mediated reduction in adiposity coincided with a lowered Firmicutes:Bacteroidetes ratio.

Soy milk has been shown to increase the relative abundance of *Lactobacillus spp*. in rats^11^. Certain *Lactobacillus spp*., including *L*. *mucosae* ^12^ and *L*. *Niu-O16* ^13^ are known equol producers that can convert daidzein into equol, as is *Enterococcus faecium* ^49^. The relative abundance of *Lactobacillus spp*. and *Enterococcus spp*. in the cecal microbiota was not altered by soy in the current study; however, confident identification of taxa was only possible at the genus level due to the limitations of the sequencing technique used. Therefore, the changes of the known equol-producing *Lactobacillus* and *Enterococcus* species are not known. The relative abundance of *Adlercreutzia*, another known equol producer that was recently identified and isolated from human feces^50^, was actually lower in the cecal microbiota of soy-fed rats. The actual abundance and activity level of *Adlercreutzia* should be further investigated. Although it is clear that gut microbiota plays a critical role in the bioavailability of isoflavones, a complete profile of equol-producing bacteria is presumably not yet identified due to the continuous discovery of many novel equol-producing bacterial strains. Correlations identified in this study suggest that the cecal bacterial taxa *Prevotella*, *Dorea*, and *Phascolarctobacterium* may provide metabolic benefits to the host. Indeed, *Prevotella* has been associated with greater fiber intake in healthy adults^51^. The gut microbiota of Hazda hunter-gatherers, which have greater microbial richness, are enriched with *Prevotella* and *Dorea* ^52^. Furthermore, *Phascolarctobacterium* is a known propionate producer^53^. Greater cecum weight suggested that there may be greater fermentation with soy supplementation. Even though the amount of total dietary fiber did not differ between two diets, soybean meal is known to contain ~8% oligosaccharides, which are highly fermentable^54^. On the other hand, the soy diet may also have lower digestibility, providing greater substrate for the gut microbiota. Microbial function and metabolites will need to be assessed to evaluate whether the gut microbiome is associated with the beneficial metabolic improvements observed in the OVX low running capacity rats.

In summary, we demonstrate that dietary soy supplementation, despite not affecting energy intake or physical activity, significantly improves insulin sensitivity and body composition of OVX rats selectively bred for low-running capacity. Furthermore, soy significantly improves blood lipid profile, adipose tissue inflammation, aortic stiffness and shifts in the cecal microbiota of rats selectively bred for low-running capacity. These findings add to the growing body of literature supporting cardiometabolic protection imparted by dietary soy consumption and extend those findings to include shifts in gut microbial health. Taken together, these findings may suggest a link between dietary soy consumption and changes within the gut microbial community that may contribute to the mechanism by which soy improves metabolic health. Further investigation is needed to elucidate mechanisms by which host-microbial interactions induced by ovariectomy and dietary soy consumption influence systemic metabolic health.

## Methods

### Animals, diet and study design

All animal procedures were approved by the Institutional Animal Care and Use Committee at the University of Missouri, Columbia. All methods were performed in accordance with the relevant guidelines and regulations. The HCR/LCR rat model was established by Drs. Britton and Koch as previously described^55^. Briefly, these rats were selectively bred based on their endurance running capacity, which was determined by running time to exhaustion on a treadmill test. Forty 27-wk-old LCR female rats (generation 32) were either ovariectomized (OVX) or sham operated (SHM) and *ad libitum* fed either soy-rich (soy) or soy-free (control) diets for 28 wk in a 2 × 2 factorial arrangement (n = 10/group): (1) OVX/soy; (2) SHM/soy; (3) OVX/control; (4) SHM/control. Rats were single-housed under controlled humidity and temperature with a 12-h light: 12-h dark cycle. Body weight (BW) and food intake were measured weekly. Intraperitoneal glucose tolerance test (IPGTT) was performed 19 wk post-surgery. At 56 wk of age, rats were euthanized via CO_2_ inhalation and exsanguination. Individual fat depots, including perigonadal (PGAT), retroperitoneal (RPAT), omental, inguinal subcutaneous (SQAT), and interscapular brown (BAT) were then dissected and immediately weighed to determine regional fat distribution, histomorphology, and gene expression. Cecal digesta was collected and snap frozen in liquid nitrogen immediately and stored in −80 °C until analysis.

### Diet formulation and analysis

Diets were purchased from Harlan Laboratories Inc. (Madison, WI), with the soy-rich diet formulated to provide an estimated 590 mg/kg diet of isoflavones [genistein and daidzein (aglycone equivalents)]. Ingredient and chemical composition of the experimental diets are reported in Table 1. Diet samples were analyzed for dry matter and organic matter according to AOAC^56^. Crude protein was determined using a Leco Nitrogen/Protein Determinator (model FP-2000, Leco Corporation, St. Joseph, MI)^56^. Fat concentrations were measured by acid hydrolysis^57^ followed by ether extraction^58^. Gross energy was measured using a bomb calorimeter (Model 1261, Parr Instruments, Moline, IL). Total dietary fiber concentrations were determined using methods described by Prosky *et al*.^59^. Total genistein, daidzein and glycitein were measured by a modification of the method of Wu *et al*.^60^ as reported in Nelson *et al*.^61^.

### Ovariectomy and sham surgeries

Surgical procedures were followed as previously described^62^. Briefly, rats were anesthetized using isoflurane. A one-inch incision was made at the midline of the dorsal surface, followed by two bilateral cuts through the muscle layer to expose the ovaries. The whole ovary, including the ovarian bursa and part of the oviduct, were removed. Sham operation was completed by externalizing the ovaries and replacing them before closing the incision using wound clips. Rats were administered acetaminophen (NSAID, 500 mg/kg) after the surgery for pain relief. OVX surgery effectiveness was determined at the conclusion of the study via verification of significant uterine atrophy.

### Intraperitoneal glucose tolerance test (IPGTT)

IPGTT was performed at 46 wk of age. Following a 6-hr fast, a baseline blood sample was taken from the tail vein at time 0. Then, an IP injection of 50% dextrose (2 g/kg BW; dose based on published protocol^63^) was administered and blood samples were collected at 15, 30, 45, 60, and 120 min post-injection for glucose concentration measurements using a handheld glucometer (AlphaTRAK, Abbott Animal Health; Chicago, IL) and insulin concentrations using a rat specific ELISA kit per the manufacturer’s instructions (Alpco Diagnostics, Salem, NH). Glucose and insulin area under the curve (AUC) calculations were performed using GraphPad Prism software (GraphPad Software, La Jolla, CA). Matsuda Index, an index that evaluates whole body insulin sensitivity, was calculated using glucose and insulin measurements throughout the IPGTT^64^.

### Adipocyte sizing

Formalin-fixed, paraffin-embedded PGAT was sectioned at 5 μm and stained with hematoxylin and eosin to determined morphology. Sections were evaluated via an Olympus BX60 photomicroscope (Olympus, Melville, NY) and images were taken at 20X magnification via SPOT Insight digital camera (Diagnostic Instruments, Sterling Heights, MI). For adipocyte cell size analysis, 100 random adipocytes from at least four fields of view, were analyzed per animal using ImageJ software (National Institutes of Health, public domain). A representative image from each group was then determined by an investigator blinded from the experimental groups.

### Intrahepatic lipid concentrations

Liver triglyceride concentrations were determined as described previously^65^. Briefly, liver samples were homogenized in 1 mL of lipid extraction solvent composed of 1:2 vol/vol methanol-chloroform and then gently agitated overnight at 4 °C. Equal part of 4 mM MgCl was added, vortexed, and centrifuged for 1 h at 1,000 × g at 4 °C. The organic phase was evaporated and reconstituted in 3:2 vol/vol butanol-Triton X-114 mix (Sigma, St. Louis, MO). Liver triglyceride concentrations were then determined using a commercially available kit (Wako L-Type TG M; Wako Pure Chemical Industries, Ltd., Osaka, Japan) and a spectrophotometer.

### Blood parameters

Fasting plasma glucose, insulin, triglyceride, total cholesterol, low-density lipoprotein cholesterol (LDL-c), high-density lipoprotein cholesterol (HDL-c), and non-esterified fatty acid (NEFA) concentrations were performed by a commercial laboratory (Comparative Clinical Pathology Services, Columbia, MO) using an Olympus AU680 automated chemistry analyzer (Beckman-Coulter, Brea, CA).

### Energy expenditure, spontaneous physical activity, and respiratory quotient

At 44 wk of age, animals were placed in metabolic chambers (PromethION; Sable Systems International, Las Vegas, NV) for a minimum of 72 hours to assess total energy expenditure (TEE), resting energy expenditure (REE), and respiratory quotient (RQ) via indirect calorimetry, and spontaneous physical activity (SPA) by the summation of x-, y-, and z-axis beam breaks. Each 72-h run captured at least two 12-hr light and two 12-hr dark cycles. Body weight and food intake were measured before and after the 72-h assessment.

### RNA extraction and RT-PCR

PGAT, BAT and liver samples were homogenized in TRIzol solution using a tissue bead homogenizer (TissueLyzer; Qiagen, Valencia, CA). Total RNA was extracted using RNeasy lipid tissue kit (Qiagen, Valencia, CA) and assessed for quality and quantity using a ND-1000 spectrophotometer (Nanodrop Technologies, Wilmington, DE). cDNA was reverse transcribed from total mRNA using the High Capacity cDNA Reverse Transcription Kit (Applied Biosystems, Foster City, CA). All the mRNA data presented were derived using the ΔΔCT approach, comparing gene expression relative to the housekeeping gene (18S); data are expressed as fold difference relative to SHM/control.

### *In vivo* aortic stiffness by pulse wave velocity

Doppler ultrasound (Indus Mouse Doppler System, Webster, TX) was used as previously described^66^ to evaluate aortic pulse wave velocity (PWV), the gold standard technique for *in vivo* determination of arterial stiffness. Prior to sacrifice, isoflurane-anesthetized mice (1.75% in 95% O_2_–5% CO_2_ stream) were placed supine on a heating board and legs secured to ECG electrodes. Determination of PWV is based on the transit time method calculated as the difference in arrival times of a Doppler pulse wave at two locations along the aorta (i.e., aortic arch and abdominal aorta at the level of renal arteries) a known distance apart measured with a ruler. Each of the pulse wave arrival times is measured as the time from the peak of the ECG R-wave to the leading foot of the pulse wave at which time velocity begins to rise at the start of systole. The distance between the two locations along the aorta is measured with a ruler and divided by transit time. Data are expressed in cm/s.

### *Ex vivo* aortic vasomotor function

Isolation and assessment of thoracic aortic ring function was determined as previously described^67^. Briefly, immediately following exsanguination, the thoracic aorta was cut into a 2-mm ring cleaned of perivascular adipose tissue and mounted in a myograph chamber (Model 620 M, Danish Myo Technology, Aarhus, Denmark) containing physiological salt solution gassed with 95% O_2_–5% CO_2_ at 37 °C. After a 30-min equilibration period, an optimal tension (~3.7 g) was applied and then another 30 min of equilibration followed. To assess viability, aortic rings were stimulated with 80KCl. Endothelium-dependent and independent relaxation was assessed with cumulative concentration-response curves to Acetylcholine (ACh, 10^−9^ to 10^−4^ M) and sodium nitroprusside (SNP, 10^−9^ to 10^−4^ M), respectively. Mounted rings were preconstricted with a submaximal concentration of phenylephrine (1 µM) prior to ACh and SNP dose-response curves. Relaxation at each concentration was measured and expressed as percent maximum relaxation, where 100% is phenylephrine.

### Cecal digesta DNA extraction, amplification, and sequencing

Total DNA from cecal digesta samples (n = 8–10/group) was extracted using Mo-Bio PowerSoil kits (MO BIO Laboratories, Inc., Carlsbad, CA). Humans are colonic fermenters whereas rodents are cecal fermenters, which means that the most active fermentation happens at different segments of the gastrointestinal tract in these two species. To best mimic humans, cecal digesta was evaluated. Concentration of extracted DNA was quantified using a Qubit® 3.0 Fluorometer (Life Technologies, Grand Island, NY). 16 S rRNA gene amplicons were generated using a Fluidigm Access Array (Fluidigm Corporation, South San Francisco, CA) in combination with Roche High Fidelity Fast Start Kit (Roche, Indianapolis, IN). The primers 515F (5′-GTGCCAGCMGCCGCGGTAA-3′) and 806R (5′-GGACTACHVGGGTWTCTAAT-3′) that target a 252 bp-fragment of V4 region were used for amplification (primers synthesized by IDT Corp., Coralville, IA)^68^. CS1 forward tag and CS2 reverse tag were added according to the Fluidigm protocol. Barcode was added to each DNA amplicon to distinguish individual samples. Quality of the amplicons was assessed using a Fragment Analyzer (Advanced Analytics, Ames, IA) to confirm amplicon regions and sizes. A DNA pool was generated by combining equimolar amounts of the amplicons from each sample. The pooled samples were then size selected on a 2% agarose E-gel (Life technologies, Grand Island, NY) and extracted using a Qiagen gel purification kit (Qiagen, Valencia, CA). Cleaned size-selected pooled products were run on an Agilent Bioanalyzer to confirm appropriate profile and average size. Illumina sequencing was performed on a MiSeq using v3 reagents (Illumina Inc., San Diego, CA) at the W. M. Keck Center for Biotechnology at the University of Illinois.

### Bioinformatics for cecal microbial analysis

Forward reads were trimmed using the FASTX-Toolkit (version 0.0.13), and QIIME 1.9.1^69^ was used to process the resulting sequence data. Briefly, high-quality (quality value ≥20) sequence data derived from the sequencing process were demultiplexed. Sequences were then clustered into operational taxonomic units (OTU) using UCLUST^70^ through a closed-reference OTU picking strategy against the Greengenes 13_8 reference database^71^ with a 97% similarity threshold. Singletons (OTUs that were observed fewer than 2 times) and OTUs that had less than 0.01% of the total observation were discarded. A total of 1,365,605 16 S rRNA-based amplicon sequences were obtained, with an average of 35,936 reads (range = 14,345–53,825) per sample. An even sampling depth (sequences per sample) of 14,345 sequences per sample was used for assessing alpha- and beta-diversity measures. Beta-diversity was calculated using weighted and unweighted UniFrac^72^ distance measures. Correlations among the relative microbial abundances and physiological parameters and gene expression data were assessed using Spearman correlation coefficient using R. Multivariate Association with Linear Models (MaAsLin) analysis was then performed using default parameters with animal ID being the random effect to assess specific taxa changes (https://huttenhower.sph.harvard.edu/maaslin).

### Statistical analysis

All data were analyzed using the Mixed Models procedure of SAS 9.3 (SAS Institute, Cary, NC) with diet and surgery being fixed effects and animal ID being a random effect. When a main effect was significant, post hoc pairwise comparisons were performed using Tukey’s multiple comparison tests. Data are reported as least-squares means ± SEM with statistical significance set as *P* < 0.05 and *P* < 0.10 considered as trends.

## Electronic supplementary material

Supplementary Table S1
