# Supplementary material for: Soy Improves Cardiometabolic Health and Cecal Microbiota in Female Low-Fit Rats
Source: Sci Rep. 2017 Aug 23;7:9261. doi: 10.1038/s41598-017-08965-0 (PMC5569109; doi:10.1038/s41598-017-08965-0)
Supplement: Supplementary file 1 — Supplementary Table S1 [file 41598_2017_8965_MOESM1_ESM.pdf]

Soy Improves Cardiometabolic Health and Cecal Microbiota in Female Low-Fit Rats

Tzu-Wen L. Cross[1,2], Terese M. Zidon[3], Rebecca J. Welly[3], Young-Min Park[3], Steven L. Britton[7,8], Lauren G. Koch[7], George E. Rottinghaus[4], Maria R. Cattai de Godoy[1,2], Jaume Padilla[3,5,6], Kelly S. Swanson[1,2], Victoria J. Vieira-Potter[3*]

Supplementary Table 1. Bacterial phyla, families, and genera of fecal samples of ovariectomized (OVX) vs. sham-operated (SHM) rats bred for low aerobic capacity fed either a soy-rich (Soy) or soy-free (Control) diet. Values are least-squared means ± SEM, n = 8-10/group. Means in a row without a common superscript letter differ, *P*<0.05.

			Soy		Control		*P* values		
**Phylum**	**Family**	**Genus**	**OVX**	**SHM**	**OVX**	**SHM**	**Diet**	**Surgery**	**Diet*Surgery**
**Firmicutes**			**65.0 ± 1.75**	**63.7 ± 1.75**	**67.8 ± 1.96**	**71.3 ± 1.75**	**<0.01**	**0.54**	**0.20**
	Undefined Clostridiales	*Undefined*	21.9 ± 2.04	27.9 ± 2.04	28.6 ± 2.28	29.5 ± 2.04	0.05	0.11	0.24
	Ruminococcaceae		18.9 ± 1.44	16.5 ± 1.44	16.8 ± 1.61	16.2 ± 1.44	0.42	0.32	0.57
		Undefined	6.95 ± 0.55	6.90 ± 0.55	6.06 ± 0.61	5.70 ± 0.55	0.07	0.72	0.77
		Oscillospira	6.13 ± 0.76	6.18 ± 0.76	7.19 ± 0.85	6.50 ± 0.76	0.38	0.68	0.64
		Ruminococcus	5.78 ± 0.72	3.42 ± 0.72	3.53 ± 0.80	3.96 ± 0.72	0.26	0.20	0.07
	Lactobacillaceae	*Lactobacillus*	10.9 ± 2.77	6.46 ± 2.77	8.24 ± 3.10	8.88 ± 2.77	0.43	0.40	0.67
	Lachnospiraceae		5.86 ± 0.80	6.26 ± 0.80	5.56 ± 0.90	4.30 ± 0.80	0.18	0.60	0.32
		Undefined	**2.62 ± 0.48**	**4.15 ± 0.48**	**1.99 ± 0.54**	**1.74 ± 0.48**	**<0.01**	**0.21**	**0.08**
		Anaerostipes	**0.03 ± 0.01**	**0.05 ± 0.01**	**0.08 ± 0.01**	**0.07 ± 0.01**	**<0.01**	**0.50**	**0.14**
		Coprococcus	1.91 ± 0.51	0.75 ± 0.51	2.43 ± 0.57	1.72 ± 0.51	0.16	0.08	0.67
		Dorea	**0.13 ± 0.03[ab]**	**0.30 ± 0.03[a]**	**0.07 ± 0.04[b]**	**0.06 ± 0.03[b]**	**<0.01**	**0.03**	**0.02**
		Roseburia	**0.01 ± 0.01**	**0.01 ± 0.01**	**0.04 ± 0.01**	**0.03 ± 0.01**	**<0.01**	**0.90**	**0.46**
		[Ruminococcus]	1.16 ± 0.19	0.99 ± 0.19	0.96 ± 0.21	0.68 ± 0.19	0.20	0.28	0.78
	Veillonellaceae		2.44 ± 0.19	1.99 ± 0.19	2.24 ± 0.22	1.95 ± 0.19	0.55	0.07	0.67
		Undefined	1.88 ± 0.23	1.56 ± 0.23	2.11 ± 0.25	1.91 ± 0.23	0.22	0.28	0.78
		Anaerovibrio	**0.30 ± 0.05**	**0.12 ± 0.05**	**0.09 ± 0.06**	**0.03 ± 0.05**	**<0.01**	**0.02**	**0.27**
		Phascolarctobacterium	0.06 ± 0.10	0.30 ± 0.10	0.00 ± 0.11	0.00 ± 0.10	0.09	0.25	0.26
		Veillonella	0.21 ± 0.10	0.01 ± 0.10	0.04 ± 0.12	0.01 ± 0.10	0.45	0.29	0.45
	Clostridiaceae		**2.20 ± 1.08**	**2.32 ± 1.08**	**3.50 ± 1.21**	**6.94 ± 1.08**	**0.01**	**0.12**	**0.15**
		Undefined	**0.64 ± 0.36[b]**	**0.12 ± 0.36[b]**	**0.87 ± 0.40[ab]**	**2.33 ± 0.36[a]**	**<0.01**	**0.22**	**0.01**
		Clostridium	0.76 ± 0.33	0.04 ± 0.33	0.57 ± 0.37	0.01 ± 0.33	0.74	0.07	0.80
		SMB53	**0.80 ± 0.67**	**2.17 ± 0.67**	**2.06 ± 0.75**	**4.60 ± 0.67**	**0.01**	**<0.01**	**0.40**
	Streptococcaceae	***Streptococcus***	**0.94 ± 0.21**	**0.89 ± 0.21**	**0.53 ± 0.24**	**0.29 ± 0.21**	**0.02**	**0.21**	**0.77**

	[Mogibacteriaceae]	*Undefined*	0.88 ± 0.25	0.66 ± 0.25	0.79 ± 0.28	1.30 ± 0.25	0.30	0.59	0.17
	Turicibacteraceae	*Turicibacter*	0.41 ± 0.13	0.28 ± 0.13	0.34 ± 0.15	0.56 ± 0.13	0.43	0.74	0.20
	Enterococcaceae	*Enterococcus*	0.25 ± 0.12	0.00 ± 0.12	0.00 ± 0.14	0.00 ± 0.12	0.35	0.35	0.35
	Peptococcaceae		0.22 ± 0.04	0.32 ± 0.04	0.22 ± 0.04	0.28 ± 0.04	0.61	0.07	0.63
		Undefined	0.11 ± 0.03	0.16 ± 0.03	0.15 ± 0.03	0.21 ± 0.03	0.13	0.05	0.93
		rc4-4	**0.11 ± 0.03**	**0.16 ± 0.03**	**0.07 ± 0.03**	**0.07 ± 0.03**	**0.02**	**0.47**	**0.41**
	Peptostreptococcaceae	*Undefined*	0.14 ± 0.26	0.20 ± 0.26	0.80 ± 0.30	0.49 ± 0.26	0.09	0.66	0.49
	Erysipelotrichaceae	*Allobaculum*	0.04 ± 0.22	0.01 ± 0.22	0.26 ± 0.25	0.59 ± 0.22	0.09	0.51	0.42
**Bacteroidetes**			**25.3 ± 1.61**	**25.5 ± 1.61**	**14.95 ± 1.80**	**14.6 ± 1.61**	**<0.01**	**0.95**	**0.86**
	Prevotellaceae		**14.3 ± 1.52**	**14.7 ± 1.52**	**4.05 ± 1.70**	**3.46 ± 1.52**	**<0.01**	**0.95**	**0.75**
		Undefined	**0.15 ± 0.03**	**0.08 ± 0.03**	**0.00 ± 0.03**	**0.00 ± 0.03**	**<0.01**	**0.22**	**0.24**
		Prevotella	**14.1 ± 1.51**	**14.6 ± 1.51**	**4.05 ± 1.69**	**3.45 ± 1.51**	**<0.01**	**0.97**	**0.74**
	S24-7	***Undefined***	**7.07 ± 0.48**	**8.85 ± 0.48**	**7.39 ± 0.54**	**8.34 ± 0.48**	**0.85**	**<0.01**	**0.41**
	[Paraprevotellaceae]		**2.23 ± 0.25**	**1.05 ± 0.25**	**1.26 ± 0.28**	**1.09 ± 0.25**	**0.09**	**0.01**	**0.06**
		CF231	**0.20 ± 0.06**	**0.16 ± 0.06**	**0.43 ± 0.07**	**0.43 ± 0.06**	**<0.01**	**0.77**	**0.84**
		Paraprevotella	0.77 ± 0.20	0.00 ± 0.20	0.00 ± 0.23	0.00 ± 0.20	0.08	0.08	0.08
		[Prevotella]	1.26 ± 0.23	0.88 ± 0.23	0.83 ± 0.26	0.66 ± 0.23	0.17	0.26	0.64
	Bacteroidaceae	*Bacteroides*	0.97 ± 0.33	0.55 ± 0.33	1.26 ± 0.37	0.44 ± 0.33	0.80	0.08	0.57
	Undefined Bacteroidales	***Undefined***	**0.47 ± 0.10^{b}**	**0.11 ± 0.10^{b}**	**0.66 ± 0.11^{a}**	**0.90 ± 0.10^{a}**	**<0.01**	**0.56**	**<0.01**
	Rikenellaceae	*Undefined*	0.23 ± 0.03	0.22 ± 0.03	0.25 ± 0.04	0.25 ± 0.03	0.53	0.89	0.89
	[Odoribacteraceae]	***Butyricimonas***	**0.04 ± 0.02**	**0.03 ± 0.02**	**0.08 ± 0.02**	**0.07 ± 0.02**	**0.04**	**0.76**	**0.99**
**Proteobacteria**			**7.53 ± 0.96^{b}**	**11.0 ± 0.96^{b}**	**14.7 ± 1.07^{a}**	**8.84 ± 0.96^{ab}**	**<0.01**	**0.23**	**0.02**
	Helicobacteraceae		**5.61 ± 0.91**	**6.56 ± 0.91**	**8.85 ± 1.01**	**7.59 ± 0.91**	**0.03**	**0.87**	**0.25**
		Undefined	**0.25 ± 0.04**	**0.30 ± 0.04**	**0.41 ± 0.05**	**0.35 ± 0.04**	**0.03**	**0.88**	**0.26**
		Flexispira	**0.18 ± 0.03**	**0.22 ± 0.03**	**0.29 ± 0.04**	**0.27 ± 0.03**	**0.03**	**0.78**	**0.39**
		Helicobacter	**5.18 ± 0.83**	**6.05 ± 0.83**	**8.16 ± 0.93**	**6.97 ± 0.83**	**0.03**	**0.86**	**0.24**
	Desulfovibrionaceae		**1.44 ± 0.57**	**2.18 ± 0.58**	**4.99 ± 0.64**	**3.40 ± 0.58**	**<0.01**	**0.48**	**0.06**
		Undefined	**1.28 ± 0.54**	**1.95 ± 0.54**	**4.60 ± 0.60**	**3.08 ± 0.54**	**<0.01**	**0.45**	**0.06**
		Desulfovibrio	**0.16 ± 0.06**	**0.23 ± 0.06**	**0.39 ± 0.06**	**0.32 ± 0.06**	**<0.01**	**0.95**	**0.22**
	Alcaligenaceae	*Sutterella*	0.37 ± 0.17	0.05 ± 0.17	0.05 ± 0.19	0.00 ± 0.17	0.31	0.30	0.45
	Enterobacteriaceae	*Undefined*	0.09 ± 0.33	0.04 ± 0.33	0.8 ± 0.37	0.00 ± 0.33	0.32	0.22	0.28
	Pasteurellaceae	***Aggregatibacter***	**0.02 ± 0.01**	**0.01 ± 0.01**	**0.02 ± 0.01**	**0.00 ± 0.01**	**0.57**	**0.02**	**0.98**
**Cyanobacteria**	***Undefined YS2***	***Undefined***	**0.81 ± 0.13**	**0.88 ± 0.13**	**0.49 ± 0.15**	**0.34 ± 0.13**	**<0.01**	**0.78**	**0.42**
**Actinobacteria**			**0.67 ± 0.34**	**0.22 ± 0.34**	**0.79 ± 0.38**	**1.60 ± 0.34**	**<0.01**	**0.44**	**0.06**
	Bifidobacteriaceae	*Bifidobacterium*	0.52 ± 0.34	0.00 ± 0.34	0.45 ± 0.38	1.3 ± 0.34	0.09	0.63	0.06

	Coriobacteriaceae		**0.14 ± 0.03**	**0.21 ± 0.03**	**0.33 ± 0.04**	**0.29 ± 0.03**	**<0.01**	**0.72**	**0.12**
		Undefined	**0.06 ± 0.02**	**0.08 ± 0.02**	**0.14 ± 0.02**	**0.09 ± 0.02**	**0.01**	**0.59**	**0.11**
		Adlercreutzia	**0.09 ± 0.03**	**0.13 ± 0.03**	**0.19 ± 0.03**	**0.19 ± 0.03**	**<0.01**	**0.49**	**0.50**
	Micrococcaceae	*Rothia*	0.01 ± 0.00	0.01 ± 0.00	0.01 ± 0.00	0.01 ± 0.00	0.12	0.96	0.47
**Spirochaetes**	***Spirochaetaceae***	***Treponema***	**0.50 ± 0.11**	**0.45 ± 0.11**	**0.88 ± 0.13**	**0.73 ± 0.11**	**<0.01**	**0.39**	**0.66**
Deferribacteres	*Deferribacteraceae*	*Mucispirillum*	0.09 ± 0.04	0.11 ± 0.04	0.18 ± 0.05	0.16 ± 0.04	0.10	0.98	0.58
**Elusimicrobia**	***Elusimicrobiaceae***	***Undefined***	**0.10 ± 0.02**	**0.05 ± 0.02**	**0.03 ± 0.02**	**0.01 ± 0.02**	**<0.01**	**0.10**	**0.49**
Verrucomicrobia	*Verrucomicrobiaceae*	*Akkermansia*	0.00 ± 0.09	0.16 ± 0.09	0.08 ± 0.10	0.28 ± 0.09	0.06	0.27	0.85
TM7	*F16*	*Undefined*	0.04 ± 0.01	0.02 ± 0.01	0.04 ± 0.01	0.03 ± 0.01	0.70	0.12	0.71
Tenericutes	*Undefined RF39*	*Undefined*	0.01 ± 0.02	0.04 ± 0.02	0.01 ± 0.02	0.01 ± 0.02	0.29	0.32	0.67

8
